# Children’s belief- and desire-reasoning in the temporoparietal junction: evidence for specialization from functional near-infrared spectroscopy

**DOI:** 10.3389/fnhum.2015.00560

**Published:** 2015-10-07

**Authors:** Lindsay C. Bowman, Ioulia Kovelman, Xiaosu Hu, Henry M. Wellman

**Affiliations:** ^1^Department of Pediatrics, Harvard Medical School, Boston, MAUSA; ^2^Division of Developmental Medicine, Laboratories of Cognitive Neuroscience, Boston Children’s Hospital, Boston, MAUSA; ^3^Department of Psychology, University of Michigan, Ann Arbor, MIUSA; ^4^Center for Human Growth and Development, University of Michigan, Ann Arbor, MIUSA

**Keywords:** theory of mind (ToM), fNIRS, temporoparietal junction (TPJ), beliefs, desires, child development, developmental cognitive neuroscience

## Abstract

Behaviorally, children’s explicit theory of mind (ToM) proceeds in a progression of mental-state understandings: developmentally, children demonstrate accurate explicit desire-reasoning *before* accurate explicit belief-reasoning. Given its robust and cross-cultural nature, we hypothesize this progression may be paced in part by maturation/specialization of the brain. Neuroimaging research demonstrates that the right temporoparietal junction (TPJ) becomes increasingly selective for ToM reasoning as children age, and as their ToM improves. But this research has narrowly focused on beliefs or on undifferentiated mental-states. A recent ERP study in children included a critical contrast to desire-reasoning, and demonstrated that right posterior potentials differentiated belief-reasoning from desire-reasoning. Taken together, the literature suggests that children’s desire-belief progression may be paced by specialization of the right TPJ for belief-reasoning specifically, beyond desire-reasoning. In the present study, we tested this hypothesis directly by examining children’s belief- and desire-reasoning using functional near-infrared spectroscopy in conjunction with structural magnetic resonance imaging to pinpoint brain activation in the right TPJ. Results showed greatest activation in the right TPJ for belief-reasoning, beyond desire-reasoning, and beyond non-mental reasoning (control). Findings replicate and critically extend prior ERP results, and provide clear evidence for a specific neural mechanism underlying children’s progression from understanding desires to understanding beliefs.

## Introduction

Theory of mind (ToM) is a complex, cognitive phenomenon. Though sometimes equated with children’s achievement of understanding false beliefs, ToM is conceptually and developmentally broader than that. Often termed a belief-desire naïve psychology, ToM involves understanding multiple causally interconnected mental concepts, and developmentally, children proceed through a progression of mental-state understandings.

A crucial, well-documented progression is that children consistently achieve an explicit understanding of desires before demonstrating an explicit understanding of beliefs (e.g., Gopnik and Slaughter, 1991; Bartsch and Wellman, 1995; Wellman and Liu, 2004). This progression holds across tasks matched on procedural methodology, as in the ‘diverse desires’ and ‘diverse beliefs’ tasks where children predict the actions of a character with preference or belief opposite to the child’s own (see Wellman and Liu, 2004 for a meta-analysis). Despite the match in task demands and format, both typically developing and socially delayed children (i.e., children with autism and deaf children born to non-signing families) consistently pass diverse-desires tasks at an earlier age than diverse-beliefs tasks (Wellman and Liu, 2004; Peterson et al., 2005), and do so across several cultures and languages (e.g., Kristen et al., 2006; Wellman et al., 2006). Though the use of implicit measures (i.e., eye-gaze) suggests some forms of belief-understanding exist prior to age 2 years, tasks requiring a deliberate verbal or pointing response strongly support a developmental progression of explicit understanding of desires before explicit understanding of beliefs.

What accounts for this progression? Given its robust and cross-cultural nature, an intriguing hypothesis is that neuromaturational factors in part underlie this development. Thus, in the present study, we use neuroscientific methods to help shed light on this important but as yet unanswered question.

Abundant studies have identified a network of brain regions consistently supporting ToM in both adults and children (e.g., Castelli et al., 2000; Gallagher et al., 2000; Saxe and Kanwisher, 2003; Saxe et al., 2009; Gweon et al., 2012; see also Carrington and Bailey, 2009; Bowman and Wellman, 2014 for reviews). In particular, neuroimaging studies demonstrate that adults exhibit a more focused and narrowed recruitment of temporoparietal junction (TPJ)—especially the right TPJ—for belief reasoning, beyond recruitment for more generalized social reasoning (Saxe and Wexler, 2005; Saxe and Powell, 2006). In children, however, there is some evidence that TPJ is less specialized compared to adults (Mosconi et al., 2005; Pfeifer et al., 2009; Sabbagh et al., 2009; Saxe et al., 2009; Gweon et al., 2012). For example, for false-belief processing (Sommer et al., 2010) and for general mental-state processing (Gweon et al., 2012), although both children and adults show similar recruitment in cortical midline regions (i.e., dorsal MPFC and dorsal anterior cingulate cortex, precuneus), additional activation occurs in the TPJ in adults but not in children (Sommer et al., 2010). Moreover, children’s right TPJ becomes increasingly selective for mental-state processing, beyond more general physical and social processing, as they age over early to middle childhood (Saxe et al., 2009), and as behavioral ToM performance improves (Gweon et al., 2012).

However, existing neuroscientific investigations of ToM have narrowly focused on belief-reasoning, or on mental-state reasoning in general, ignoring critical comparisons to desire-reasoning which are central to uncovering neural mechanisms underlying children’s progressive mental-state understandings as evidenced in the behavioral data. Only one recent study has directly examined the neural correlates of belief- and desire-reasoning in children. Bowman et al. (2012) recorded ERPs when 7- and 8-years-old performed diverse-desires and diverse-beliefs tasks, expanding on the work of Liu et al. (2009) who used the same tasks and methods with adults. As a control, participants performed parallel non-mental, diverse-physical tasks (requiring reasoning about where different things go). For both adults and children, results revealed two neural systems for belief- and desire-reasoning: one associated with mid-frontal scalp regions in which potentials for belief- and desire-reasoning equally differentiated from potentials for the physical control, but not from each other, and another associated with right-posterior scalp regions in which potentials for belief- and desire-reasoning critically differed. In children, this right posterior belief-desires distinction emerged only when children exhibited accurate belief-reasoning.

These ERP results, in conjunction with neuroimaging findings demonstrating developmental specialization of the right TPJ, suggest a straightforward but important developmental possibility: there are developing specializations within TPJ recruited for specifically belief-reasoning over and above reasoning about desires, and this specialization may pace, in part, children’s progression from explicit understanding of desires to explicit understanding of beliefs (Wellman and Liu, 2004).

The present study tests this hypothesis directly. The low spatial resolution of ERP methods leaves unknown whether the “right posterior” findings from Bowman et al. (2012) actually correspond to the right TPJ. Thus here we provide needed validation by obtaining converging and neuroanatomically specified measurements of children’s brain activity for the same diverse-belief and diverse-desire tasks as used in the Bowman et al. (2012) ERP study, adjusted for hemodynamic data collection via functional near-infrared spectroscopy (fNIRS). fNIRS facilitates pinpointing of brain activation in specific cortical regions of interest (ROI), and thus via ROI analyses, can address the central question of whether children’s right TPJ exhibits specialization for belief-reasoning beyond desire-reasoning.

Functional magnetic resonance imaging (fMRI) could also provide the needed localized activation data, but we opted for fNIRS because it is more child friendly. Though localization accuracy is not as high as fMRI, fNIRS is less susceptible to movement artifact, is quiet, and does not require confinement to a narrow tube during testing—factors helpful for collecting data from younger children, especially when administering tasks with large numbers of trials as in the present study (e.g., see Brink et al., 2011 for fNIRS study examining cognitive and affective empathy in children 4- to 8-years-old). One drawback of fNIRS is that it can record activity in only the surface layers of cortex; it cannot penetrate to deeper, medial regions (Huppert et al., 2009), especially medial-frontal regions (Cooper et al., 2012). Fortunately, right TPJ lies in the surface layers of the right posterior cortex, making fNIRS suitable for examining the focal hypothesis for this study, which concerns early development and specialization of specifically the right TPJ.

We conducted an ROI analysis targeting the fNIRS channels overlaying the right TPJ. We hypothesized greater activity in these channels during children’s belief-reasoning compared to their desire-reasoning, and compared to non-mental reasoning (physical control condition), which would critically replicate and extend ERP findings from Bowman et al. (2012), linking them to broader neuroimaging literature, and providing clear evidence that children’s developmental progression from desire- to belief-understanding may be paced in part by a specialization the right TPJ for belief-reasoning. We also targeted left TPJ given adult and child neuroimaging studies also implicate this region in ToM. We hypothesized left TPJ may also show greatest activation to belief-reasoning, in line with the common pattern of more diffuse/bilateral neural specialization observed in early versus later development (Casey et al., 2000). Finally, we recorded from the anterior frontal cortex (AFC), which is not implicated in the ToM neural network, and therefore should not show activation for mental-state reasoning beyond a non-mental control. Data from this AFC ROI thus served as important contrast to our focal TPJ ROIs, to ensure data from those focal ROIs did not simply reflect changes in systemic blood flow from scalp or other tissues that lie above the cortex (Boas et al., 2004; Aslin, 2012).

## Materials and Methods

### Participants

Twenty-one typically developing children (13 males) ages 6- to 10-years-old participated in the study. All recruitment and collection procedures complied with our institution’s ethics review board. Parents gave written informed consent and children gave verbal informed assent prior to participation in accordance with the Declaration of Helsinki. Data collection stopped when the fNIRS system (on temporary loan to our institution) was no longer available. All participants were right handed, with normal or corrected-to-normal vision. Performance on standardized verbal and non-verbal intelligence tests from the Kaufman Brief Intelligence Test 2 (KBIT-2; Kaufman and Kaufman, 1990) confirmed this sample had age- and grade-appropriate verbal and non-verbal IQ abilities (verbal IQ: *M* = 29, *SD* = 7.58; non-verbal IQ: *M* = 28.64, *SD* = 5.66). Ten children were excluded from final data analysis: four due to equipment malfunction; five who did not pass the data artifact criteria (see imaging methods below); and one due to below-chance performance across all conditions indicating inattention to the task. The final sample consisted of 11 children (age range: 74–129 months; *M* = 92.9, *SD* = 15.84; nine males). Importantly, children in this sample were drawn from the same geographic area as that of the Bowman et al. (2012) ERP study, and the ages of that sample and this one did not differ statistically [*t*(28) = -1.03, *p* = 0.278].

### Measures and Procedure

#### fNIRS Tasks

We used the same tasks as Bowman et al. (2012): multi-trial diverse-desires, diverse-beliefs, and diverse-physical judgment (as control). Only the duration of the trial phases and the overall block structure were changed to optimally capture the hemodynamic response measured by fNIRS.

**FIGURE 1:**
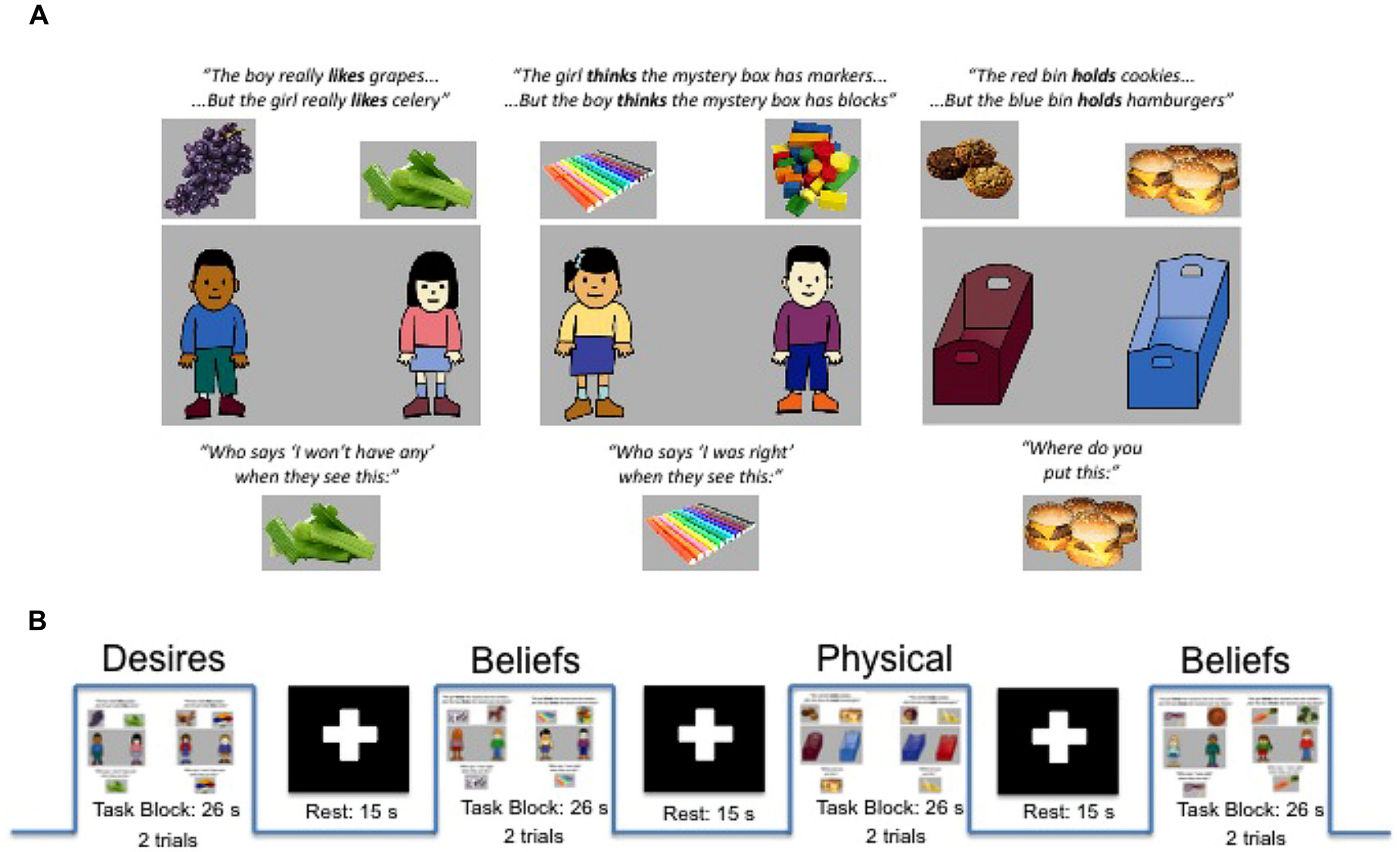
**(A)** Examples of single trials for Diverse-Desires condition (left), Diverse-Beliefs condition (middle), and Diverse-Physical condition (right) with examples of information phase (top) and target questions (bottom) as well as sample graphics for both food and toy trial types. **(B)** Schematic of experimental and rest blocks as presented within a run. Adapted from Bowman et al. (2012).

In each trial, for each of the three conditions, the participant heard a recorded female voice aurally present information about (a) a boy and girl with diverse desires (e.g., the boy likes apples but the girl likes grapes) for the Desires condition, (b) a boy and girl with diverse beliefs (e.g., the boy thinks the box has apples but the girl thinks the box has grapes) for the Beliefs condition, or (c) two bins that each held different things (e.g., the red bin holds apples but the blue bin holds grapes) for the Physical condition. At the end of the trial, participants heard a corresponding target question: (a) For Desires: “Who says ‘I’ll have some’ when they see this?”/“Who says ‘I won’t have any’ when they see this?” (when the story was about food), or “Who says ‘I’ll play with it’ when they see this?”/“Who says ‘I won’t play with it’ when they see this?” (when the story was about toys). (b) For Beliefs: “Who says ‘I was right’ when they see this?” or “Who says ‘I was wrong’ when they see this?” And (c) for the Physical condition: “Where do you put this?” or “Where do you not put this?” **Figure 1A** provides a schematic of task conditions (for full description, see Bowman et al., 2012).

The trial type (food or toys) as well as the question presented (positive or negative wording) were randomized in each trial and balanced across conditions and runs. After the target question, participants immediately saw a picture of one of the two foods/toys (e.g., apples). After seeing the revealed food/toy, the boy and girl (for Desires/Beliefs conditions) or two bins (for Physical condition) reappeared on screen, and participants were then allowed to answer by choosing one of the two characters/bins via button press. Because trials for all three conditions were constructed to have the same perceptual and linguistic structure, any differences between conditions point to differences in processing the content of the questions—belief-processing, desire-processing, or non-mental processing—beyond these perceptual and task similarities.

The task was presented in three runs of 10 experimental blocks (26 s per block) and nine rest blocks (15 s per block); these durations are within the range of other block designs capturing hemodynamic response in children with both fMRI (e.g., Gweon et al., 2012) and fNIRS (Minagawa-Kawai et al., 2011). Experimental and rest blocks alternated, with the experimental block beginning and ending each run. Each experimental block consisted of two trials (13 s per trial; 8 s for information phase plus 1.5 s for target image duration, plus 3.5 s allowed for the target questions and participant response), and trials within a block were always of the same condition (e.g., two Physical trials or two Beliefs trials). See **Figure 1B** for task and block structure.

Each condition type occurred in three blocks in two of the runs, and four blocks in one of the runs. Order of condition blocks was randomized except that no one condition type repeated successively. At the end of each run (6.6 min in duration), participants could take a small break. Total experiment time including breaks was ~25–30 min. Participants were instructed on how to do the fNIRS task immediately prior to data acquisition, and completed a practice task that went through an example trial from each of the three conditions before beginning the experiment.

#### Behavioral Tasks

Standardized verbal and non-verbal intelligence tests from the KBIT-2 (Kaufman and Kaufman, 1990) were administered after the fNIRS imaging. All participants performed at age- and grade-appropriate levels.

### fNIRS Imaging

#### Data Acquisition and Procedure

**FIGURE 2:**
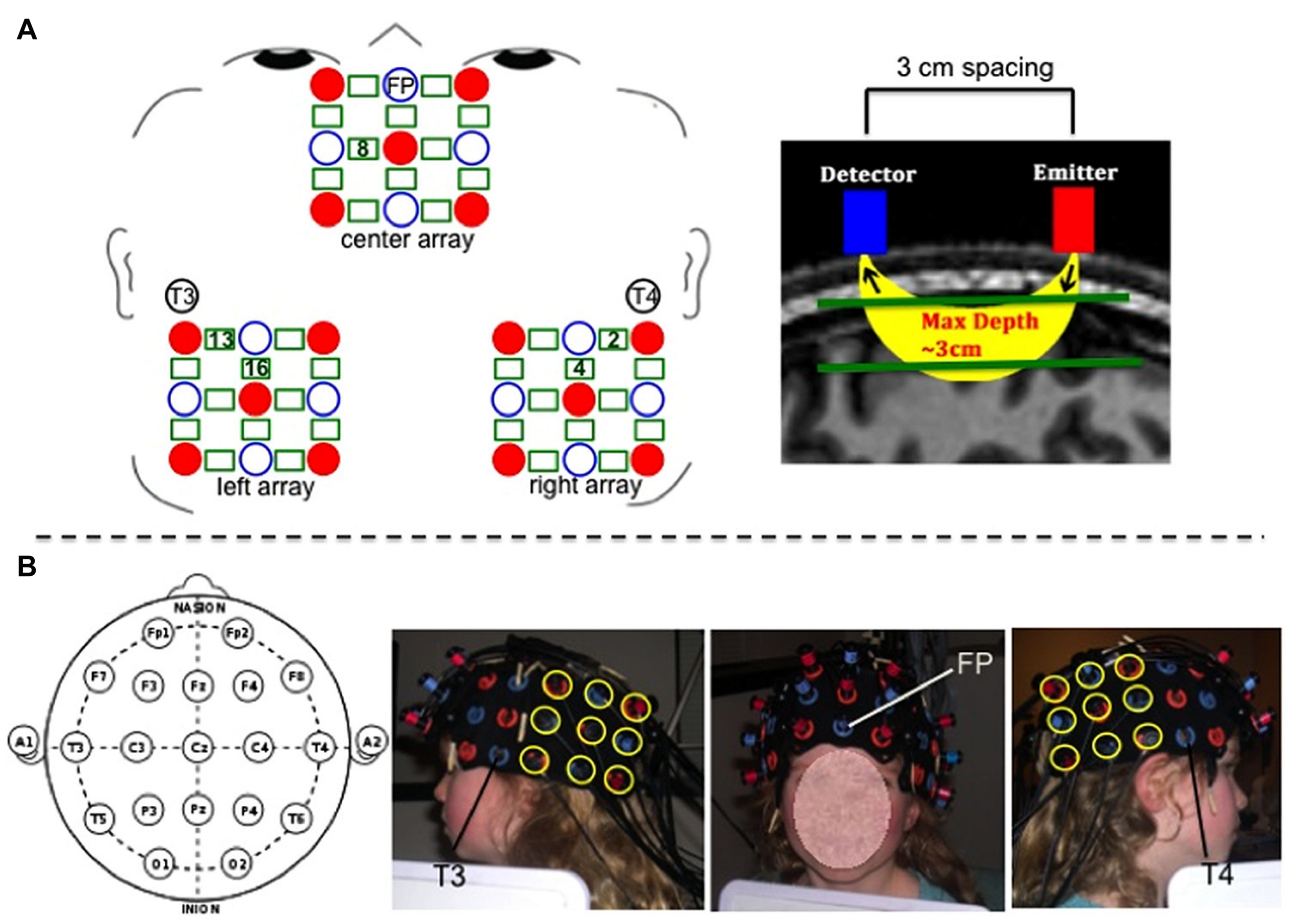
**(A**-left) Schematic of center, left, and right optode arrays showing emitters as red solid circles, detectors as blue open circles, and channels as green rectangles. 10–20 landmarks corresponding to specific optode placements are labeled. Green numbers within rectangles mark the positions of the specific channels used for analyses. **(A-right)** Depiction of spacing between detector and emitter optodes and channel of near-infrared light penetrating 3 cm through cortex. **(B)** Photographs of optode placement on child participant guided by 10–20 landmarks. For the center array, the center optode in the most ventral row was placed over coordinate Fp, and the center optode in the most dorsal row was placed in line with coordinate Fz. For the left and right arrays, the most ventral anterior optode was placed 3 cm posterior to coordinate T3 (for left side) and T4 (for right side), and the most dorsal anterior optode was positioned so as to be 3 cm posterior to the T3–C3 line (for left side) and T4–C4 line (for right side).

Hemodynamic response was recorded using a Hitachi ETG-4000 with 48 channels acquiring data at 10 Hz. A subset of 36 channels was used for the present study. The near infrared lasers (emitter optodes) were factory set to 690 and 830 nm. Optodes were segregated into three 3 × 3 arrays each containing five emitters and four detectors to create 12 channels per array (a channel is defined as the curve of near-infrared light traveling between the emitter and detector from which the hemodynamic response is measured; see **Figure 2**). Optode separation was 3 cm.

Three 9-optode arrays (center, left, and right) were secured on participants’ heads using custom-made fabric ties, and positioned according to the 10–20 system using Fp, T3, and T4 coordinates as placement landmarks to maximally overlay the AFC (center array), and the left and right TPJ (left and right arrays). See **Figure 2** for placement details. We also conducted magnetic resonance imaging (MRI) scanning to verify and further clarify the channels’ neuroanatomical positioning. Photographs were taken of the secured optode positions before and after children completed the fNIRS task to ensure that the optodes did not move over the course of the experiment.

#### ROI Identification: MRI Coregristration

Separate from the fNIRS imaging and behavioral testing session, an MRI anatomical scan was collected from one child representative of the sample (typically developing male, age: 102 months). Three 3 × 3 arrays of vitamin E tablets were constructed to exactly mimic the optode arrays (i.e., tablets arranged in same geometrical structure and situated 3 cm apart). These arrays were positioned on the child’s head using the same 10–20 coordinates as used to position the optodes (a method discussed in the review by Tsuzuki and Dan, 2014) and were secured in place using MRI-safe tape and wraps. Then, a T1-weighted Fast Spoiled Gradient Echo scan was conducted to obtain the high resolution MRI anatomical image (43 sagittal slices, slice thickness = 3 mm, TE = 5.7 ms, TR = 250 ms, flip angle = 90°, bandwidth = 15.63, F.O.V. = 22 cm). Data were collected on a three Tesla scanner at the university’s fMRI laboratory facility, while the child watched a short, silent cartoon video.

**FIGURE 3:**
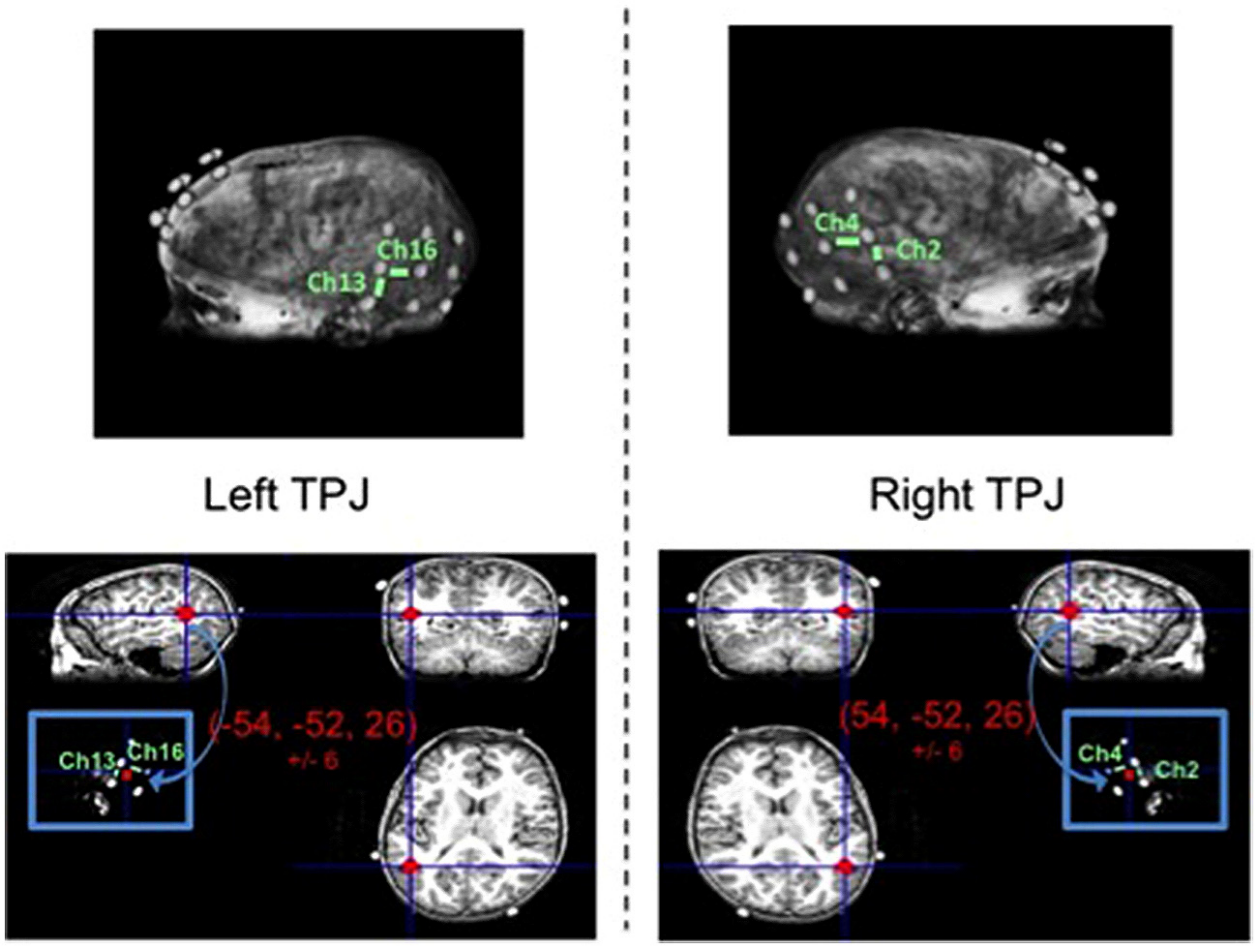
**Magnetic resonance imaging (MRI) anatomical scan of representative child participant with vitamin E probes secured to head mimicking optode positions (top left and right).** Focal channels of interest are labeled for the left TPJ (top left) and right TPJ (top right). Bottom panel depicts the TPJ regions of interest (red circles) that include the range of MNI coordinates for left TPJ (bottom left) and right TPJ (bottom right) as identified in a separate theory-of-mind fMRI study with children of similar ages to the present study.

The anatomical image was used to identify the specific fNIRS channels overlaying the left TPJ (LTPJ) and right TPJ (RTPJ) focal ROIs for analysis in our child sample. The process of coregistering fNIRS optodes with MRI structural scans to identify underlying cortical areas has been previously published with pediatric samples (e.g., Lloyd-Fox et al., 2014). The process of MRI co-registration for the present study was as follows. MNI coordinates for LTPJ and RTPJ were taken from a separate fMRI study that examined ToM reasoning in 12 comparably aged typically developing children, 7- and 8-years-old (Bowman et al., in preparation; see also Gweon et al., 2012). This fMRI study defined LTPJ and RTPJ ROI based on a combination of anatomical information (from separate localizer tasks as used in previous literature, e.g., Saxe and Kanwisher, 2003; Saxe et al., 2009) and functional activation from a ‘mental-state condition > physical control condition’ contrast—an approach to ROI definition that has been validated in previous pediatric fMRI examinations that used similar ToM tasks (Saxe et al., 2009; Gweon et al., 2012). The grand average and standard deviation for the left and right TPJ ROIs were calculated for this sample of 12 children: -54, -52, 26, ±6 for LTPJ and 54, -52, 26, ±6 for RTPJ. For the present study, these ranges of coordinates were used to define reasonably broad left and right TPJ ROIs in the single child’s MRI anatomical scan, in order to identify which particular fNIRS channels were positioned over the left and right TPJ. Specifically, using MRIcro software (http://www.mccauslandcenter.sc.edu/mricro/mricro/mricro.html), we marked on the child’s structural scan a reasonably broad ROI surrounding the mean MNI coordinates from the age-matched sample, based on the sample standard deviation (i.e., -54, -52, 26, ±6 for LTPJ and 54, -52, 26, ±6). We then determined which fNIRS channels (given their 3 cm penetration arch between emitter and detector) overlaid these ROIs, given the vitamin E placement which critically mimicked the fNIRS optode emitter and detectors. This process revealed that two channels penetrated our ROI (channels 2 and 4 on the right and channels 13 and 16 on the left). Thus data from these channels were taken as pinpointing the right and left TPJ ROIs, and were used to guide the ROI analyses (see **Figure 3**).

#### fNIRS Data Processing and Analyses

Functional near-infrared spectroscopy data were exported and analyzed using custom software in Matlab (The MathWorks, Inc.) validated in previous fNIRS studies (e.g., Kovelman et al., 2009, 2011, 2012, 2014; Shalinsky et al., 2009), and in line with diffuse optical imaging principles (Boas et al., 2004; Huppert et al., 2009). In brief, after the recording session, data were exported and analyzed using MATLAB-based software (we thank Dr. Laura Ann Petitto for making this software available to us, for details see Kovelman et al., 2009). First, the raw time course data were converted into units of optical density change (ΔOD). The data were further band-pass filtered in the range between 0.01 and 0.8 Hz. This band-pass filter was selected *a priori*, based on previous research. Specifically, the normalized intensity was band-pass filtered using third-order Butterworth filters with 0.8 and 0.01 Hz cutoff frequencies, respectively. The low pass filter value is designed to remove mainly measurement noise and cardiac physiological noise (>0.8 Hz), while the high pass filter value removes slow drift caused by motion or optode slow shifting (<0.01 Hz). These filter values are on par with other studies which have used low-pass cut-off frequencies from 0.1 Hz (Naito et al., 2007) to 1.25 Hz (Zhang et al., 2007). High-pass cut-off frequencies in other studies include 0.004 Hz (Zhang et al., 2007) and 0.01 Hz (Gratton et al., 2006). Participants’ data were baseline corrected by subtracting out the mean intensity of the optical signal recorded during the 15 s rest periods. The hemoglobin concentration change data (C_HbO_ and C_HbR_) were calculated using the modified Beer–Lambert law (mBL; Delpy et al., 1988), yielding HbO (oxygenated hemoglobin) and HbR (deoxygenated hemoglobin) values (see Huppert et al., 2009 for details). Specifically, the signal was first converted to optical density change using the following equation: ΔOD = -log $\left(\frac{I_{t}}{I_{o}}\right)$. Then the ΔOD went through the motion correction process, and was band-pass filtered. Finally, the processed data were converted into HbO and HbR changes via the mBL law:

$$\Delta OD_{\lambda_{1}}=(\varepsilon_{\lambda_{1}}^{HbO}\cdot \Delta C_{HbO}+\varepsilon_{\lambda_{1}}^{HbR}\cdot \Delta C_{Hb})\cdot DPF_{\lambda_{1}}\cdot L$$

$$\Delta OD_{\lambda_{2}}=(\varepsilon_{\lambda_{2}}^{HbO}\cdot \Delta C_{HbO}+\varepsilon_{\lambda_{2}}^{HbR}\cdot \Delta C_{Hb})\cdot DPF_{\lambda_{2}}\cdot L$$

The calculated HbO and HbR concentration changes are considered to be percent signal change (PSC).

The time course data (all channels, all conditions) for each participant were plotted in Matlab and visually inspected for motion artifacts and signal quality. Specifically, coders visually inspected data in all channels, in every block, for all runs, for all participants. Artifacts were identified as any portions of data in which signal change occurred over a period of time that was too fast to be physiological (specifically, a change in magnitude of response >0.2 and occurring in less than 3 s). Coders first identified portions of data that they judged to likely meet criteria for artifact, and then confirmed by calculating the magnitude and time period of that portion of the data. If criteria were met, the channel, block, run, and participant number were recorded and flagged for removal. Likewise, individual channels that showed activation at either >5 or 0 were also removed. Finally, any block in which 10 channels were bad were also removed. Only data that met these criteria were removed. Given the subjectivity of initially identifying portions of artifact, inspection was done by one primary coder, with two additional coders inspecting 20% of the data to ensure reliability. This method of artifact rejection is in line with prior published fNIRS work examining child data (e.g., Kovelman et al., 2012). In total, the percentage of blocks containing minimal or no artifact that were thus retained for analyses was 96.4% for the Beliefs condition, 98.2% for the Desires condition, and 99.4% for the Physical condition.

**FIGURE 4:**
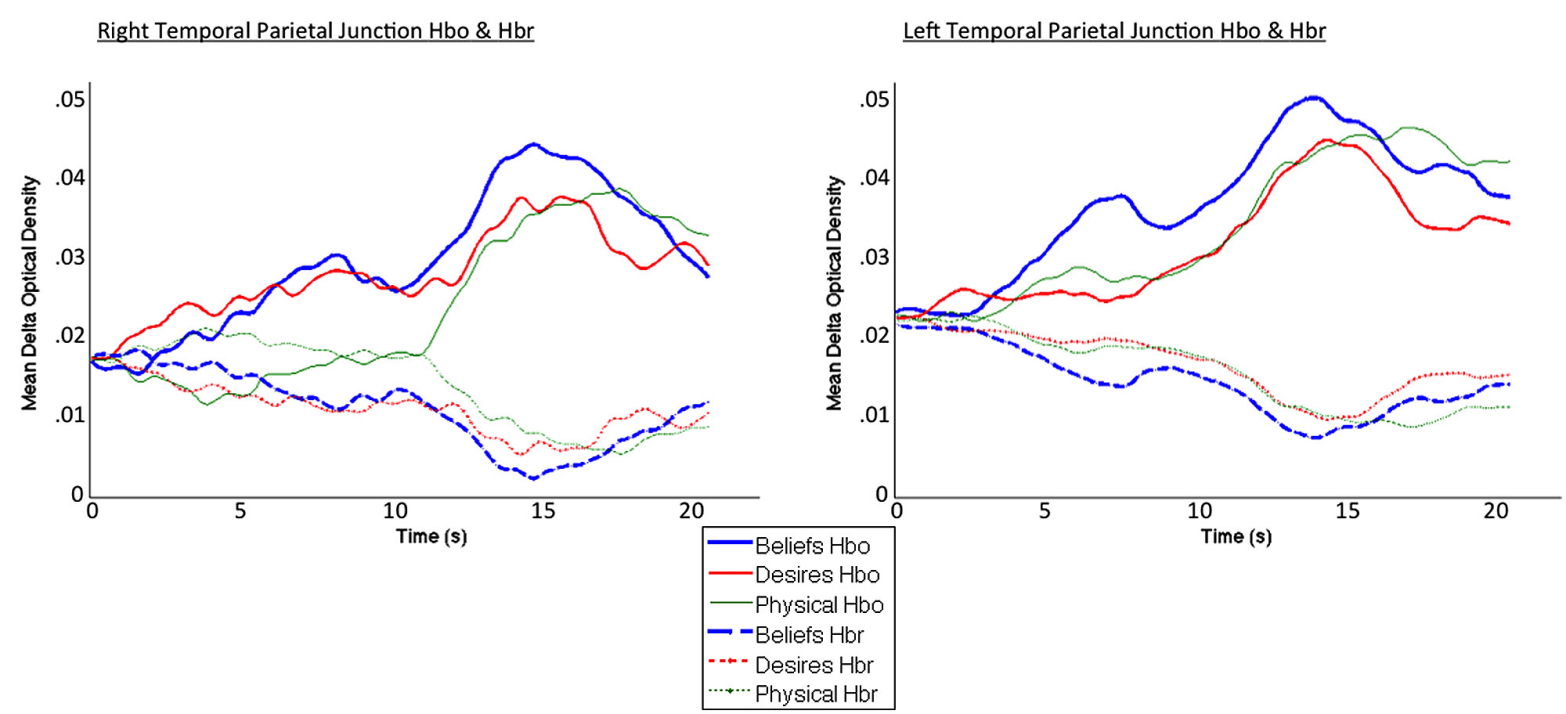
**Averaged HbO (solid lines) and HbR (dotted lines) time course for beliefs (blue thick line), desires (red thinner line), and physical (green thinnest line) conditions.** Curves exhibit the canonical slow surge in oxy-hemoglobin with simultaneous decrease in deoxy-hemoglobin over the course of the experimental block, in the 0–20 s window selected for analysis (total block duration = 26 s).

Coders also visually inspected individual participant hemodynamic response plots to identify epochs of clear neural activity from which to extract oxy hemoglobin values for focal analyses. Specifically, for each participant, in each block, coders—blind to condition, focal channels/ROIs, and study hypotheses (inspected data were collapsed across condition, and across all channels in a given probe set)—inspected oxy and deoxy plots beginning 1 s prior to block onset, across the entire 26-s block, and up to 4 s post-block termination, to identify the windows of time in which the oxy signal rose and began to fall, accompanied by a deoxy signal down-sweep and beginning rise. Coders were given Figure 6A in the Boas et al. (2004) paper as a canonical pattern to identify (focusing on the oxy and deoxy curves). Given the subjectivity involved in this visual inspection, coding was again done by one primary coder with two additional coders ensuring reliability. A similar method of window identification has been used in previously published fNIRS research (Kovelman et al., 2008, 2009, 2012, 2014). For the left and right focal arrays, the window of 0–20 s post-experimental block onset captured the canonical hemodynamic response curves indicative of brain activity (Boas et al., 2004). Thus, this window was used to extract mean oxy-hemoglobin values for final analyses. See **Figure 4** for HbO and HbR plots across the 0–20 s window, for each of the three conditions.

To summarize, within the 0–20 s window, percent signal change in oxy-hemoglobin (HbO) values were averaged across blocks of the same condition type, across all usable participant data, to obtain a grand average mean HbO response for each of the three conditions. HbO constitutes a far greater portion of signal form the cortex (76%) compared to HbR (19%; Gagnon et al., 2012). Moreover, HbO is sampled over a larger region of brain tissue, and the signal-to-noise contrast for HbO is better than HbR (Strangman et al., 2002). The correlations between the canonical model of the hemodynamic response function and models of HbO (versus HbR) are consistently higher (Huppert et al., 2009). Thus, we used HbO as a more robust index of underlying neural activity. This grand mean HbO response was then compared across Beliefs, Desires, and Physical conditions to examine the pattern of brain activity across the two ROIs—right channels 2 and 4 (RTPJ), and left channels 13 and 16 (LTPJ)—and in a contrast control center channel overlaying the AFC.

For our focal TPJ ROIs, as is commonly done in ROI analyses (e.g., Saxe and Kanwisher, 2003), we conducted both a group-level ROI as well as an individual-level ROI analysis, to provide a fuller picture of the differences in brain activation across conditions. For the group ROI analysis, we averaged each participant’s data from channels 2 and 4 on the right, and channels 13 and 16 on the left, to create left and right TPJ ROIs for each participant. For the individual ROI analysis, the ROI selection criterion was based on an overall general mental-state activation (averaged across Beliefs and Desires conditions) versus physical activation pattern. Thus, significant Desire > Physical and Belief > Physical results are more likely, given the functional definition for the individual ROIs rests on a “Mental” (i.e., Belief and Desire average) > Physical criterion. Critically though, this functional criterion yields a more conservative approach to investigating crucial differences between the two focal mental conditions—i.e., Belief versus Desire—because it directs where to look for such distinctions based on a the combined mental—i.e., Beliefs *and* Desires—versus non-mental criterion. It is the investigation of these beliefs-desires differences that are central to the study.

Specifically, to create individual RTPJ ROIs, we averaged across Beliefs and Desires conditions, in each of the right channels separately (i.e., ch2 and 4, separately) to calculate the average ‘mental-state activation’ in each of those channels, for each child. We then conducted per-channel comparisons of children’s brain activation during this mental-state condition versus their activation during the Physical condition (e.g., ch2 Mental-state verses ch2 Physical), and selected the channel (i.e., 2 or 4) that demonstrated the greatest Mental > Physical activation difference to represent each child’s individual RTPJ ROI. The following selection criteria were employed. For each child, Mental mean oxy had to be significantly greater (at *p* < 0.001) across the 0–20 s window than Physical mean oxy. If both channels met Mental > Physical significance criterion, oxy signal was averaged across the 0–20 s window, Physical activation was subtracted from Mental activation in each contending channel, and the channel yielding the greatest positive value was selected. No child had identical activation differences in each channel, and children without a Mental > Physical pattern across either channel were excluded from the individual ROI analysis. We repeated these steps for channels 13 and 16 to define individual LTPJ ROIs. For RTPJ, 8/11 children were included in analyses (five children with a ch2 ROI and three with ch4 ROI). For LTPJ, 7/11 children were included in analyses (one child with a ch13 ROI and six with ch16 ROI). This process is in line with existing ROI selection processes for fMRI investigations of ToM in children that also target regions of maximal mental versus non-mental differences as part of the individual ROI selection criterion (Saxe et al., 2009; Gweon et al., 2012). Given that the AFC ROI was analyzed solely as contrast for these focal TPJ ROIs, one channel was selected from the AFC to serve as this contrast, and thus no individual ROI analyses were carried out.

## Results

### fNIRS Task Performance Accuracy

As expected, children were better at solving diverse-desires (90.3% correct) and physical control tasks (87.9%) compared to diverse-beliefs (62.3%); Beliefs condition versus Desires and Physical conditions, *t*(10) = -6.07, *p <* 0.001, *t*(10) = -6.93, *p* < 0.001, respectively. Desires and Physical conditions did not differ from each other, *t*(10) = 0.91, *p* = 0.385. This pattern is identical to performance accuracy on the parallel ERP task in Bowman et al. (2012), and is consistent with numerous findings demonstrating that, compared to desire-understanding, belief-understanding emerges later in childhood (see meta-analysis in Wellman and Liu, 2004), and can be less accurate/fluent in older children and adults (e.g., Malle, 2004). Neither accuracy in any condition, nor age of participant were related to mean HbO in any ROI, for either group or individual ROI analyses (all *rs* < 0.27, *p*s > 0.42). Thus, neither age nor performance accuracy were considered further in analyses with HbO.

### Brain Activation

For all ROI analyses below we adopted a traditional alpha of *p* < 0.05, but we also considered results marginally significant at *p* < 0.1.

#### Group ROI Analyses: Right and Left TPJ

Results of the MRI anatomical scan implicated more than one fNIRS channel as covering the RTPJ and LTPJ ROIs, and so we averaged data across channels 2 and 4 (right side) and 13 and 16 (left side) to create group right and left TPJ ROIs, respectively.

**FIGURE 5:**
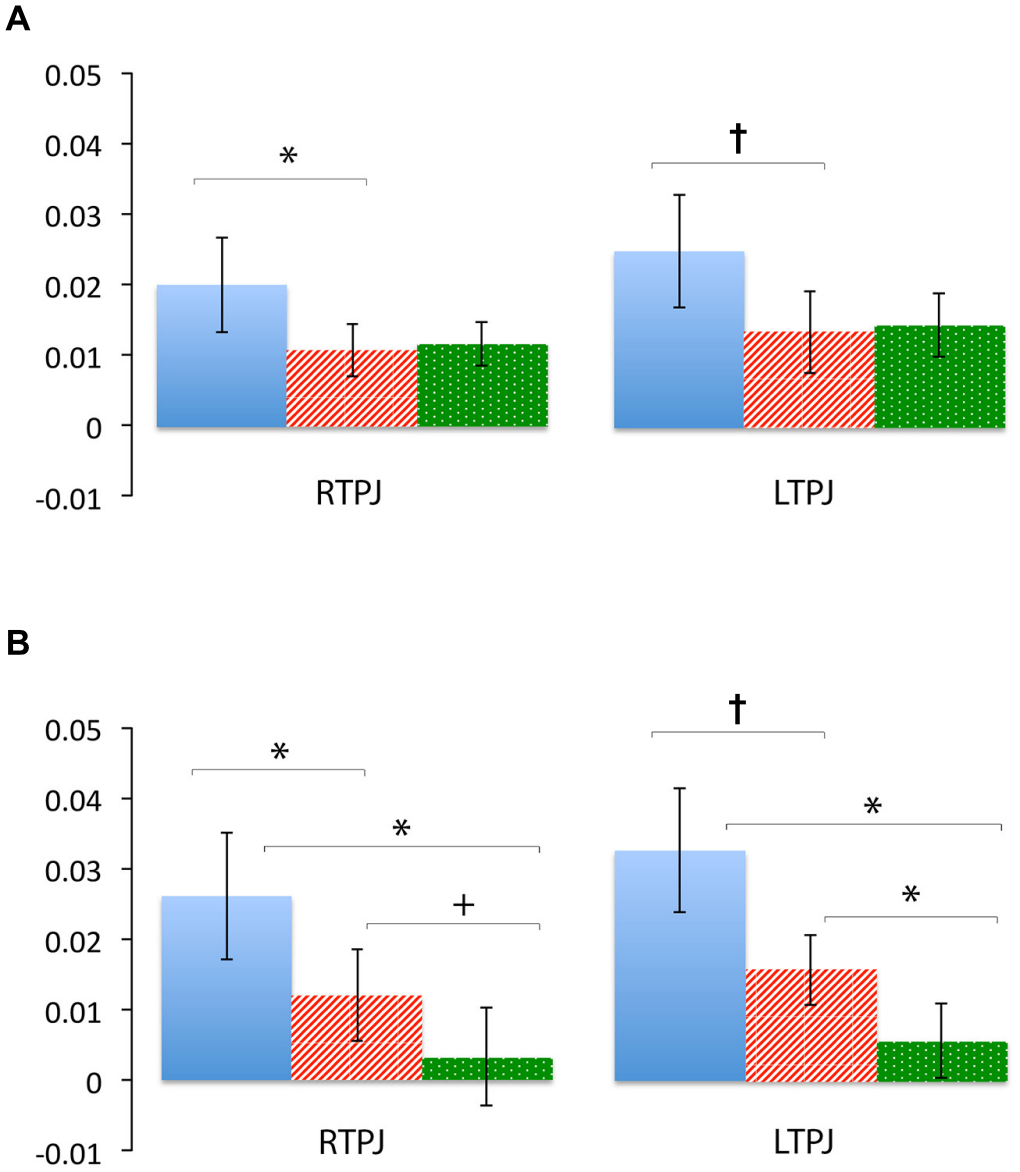
**Percent mean oxy (HbO) signal change for Beliefs conditions (blue solid), Desires conditions (red striped), and Physical conditions (green dotted) in LTPJ and RTPJ for the group ROI analyses **(A)**, and the individual ROI analyses with ‘Mental > Physical’ inclusion criteria (B).** Results indicate specialization for belief-reasoning (over desire-reasoning) in the RTPJ as evidenced by significantly greater oxy signal for beliefs versus desires conditions across both analyses. Results also suggest some evidence for belief-specialization in the LTPJ, though effects are less robust.*p < 0.05, *p*† < 0.1.

**Figure 4** demonstrates clearly greater mean HbO for the Beliefs compared to the Desires and Physical conditions across the analysis window, for both left and right TPJ. Indeed, omnibus repeated measures ANOVAs comparing mean HbO activation (averaged over each left and right channel pair) across Beliefs, Desires, and Physical conditions revealed a significant condition effect in the RTPJ [*F*(2) = 3.67, *p* = 0.043], and a marginally significant effect in the LTPJ [*F*(2) = 2.65, *p* = 0.096]. Results of follow-up paired-samples *t*-tests are shown in **Figure 5A** and the top panel of **Table 1**. For both RTPJ and LTPJ, mean HbO was greater for Beliefs compared to both Desires and Physical conditions. The Beliefs-Desires difference was significant for RTPJ, and marginally significant for LTPJ. In short, RTPJ shows differential brain response to the demands of the Belief versus Desire conditions, with belief-activation *greater* than desire-activation. A similar but less robust Beliefs-Desires distinction exists in LTPJ.

**Table 1:** Paired-samples *t*-tests comparing children’s mean oxy-hemoglobin response in the three condition contrasts in left and right TPJ for group and individual ROI analyses.

Comparison	RTPJ	LTPJ
**Group ROI (Channel-pair Average**)		
Belief versus Desire	*t*(10) = 2.32, *p* = 0.043*	*t*(10) = 1.87, *p* = 0.091†
Belief versus Physical	*t*(10) = 1.75, *p* = 0.110	*t*(10) = 1.56, *p* = 0.149
Desire versus Physical	*t*(10) = -0.46, *p* = 0.653	*t*(10) = -0.33, *p* = 0.749
**Individual ROI (Mental > Physical)**		
Belief versus Desire	*t*(7) = 2.42, *p* = 0.046*	*t*(6) = 2.21, *p* = 0.069†
Belief versus Physical	*t*(7) = 2.78, *p* = 0.027*	*t*(6) = 3.24, *p* = 0.018*
Desire versus Physical	*t*(7) = 2.16, *p* = 0.068†	*t*(6) = 4.01, *p* = 0.007*

*Indicates significance at *p* = 0.05;† indicates significance at *p* = 0.1.

#### Individual ROI Analysis: Right and Left TPJ

To take account of possible heterogeneity in ROIs’ functional organization across participants, we also conducted an individual ROI analysis by analyzing data from *either* channel 2 or 4 (on the right), and *either* channel 13 or 16 (on the left) for each participant. See methods for full individual ROI selection details.

As shown in **Figure 5B**, for both RTPJ and LTPJ ROIs, the predicted pattern of participants’ activation for Beliefs greater than activation for both Desires and Physical conditions was again demonstrated. In line with findings from the group ROI analyses, in RTPJ, mean activation for Beliefs was significantly greater than for Desires, and in LTPJ, the Beliefs-Desires distinction was again only marginally significant (see bottom panel of **Table 1** for statistics). More clearly than in the group ROI analyses, and as expected given the function individual ROI criterion of mental > physical channels, these analyses revealed that participants’ activation for Beliefs was significantly greater than *Physical* activation in both RTPJ and LTPJ. Moreover, participants’ Desires activation was greater than Physical activation in RTPJ and LTPJ as well.

These results replicate the finding from the group ROI analysis that the TPJ is recruited for belief-reasoning, over and above recruitment for desire-reasoning, with the effect demonstrated most robustly in the RTPJ. The Beliefs > Physical findings also demonstrate TPJ specialization for processing beliefs, beyond non-mental processing more generally.

#### Contrast AFC ROI

**FIGURE 6:**
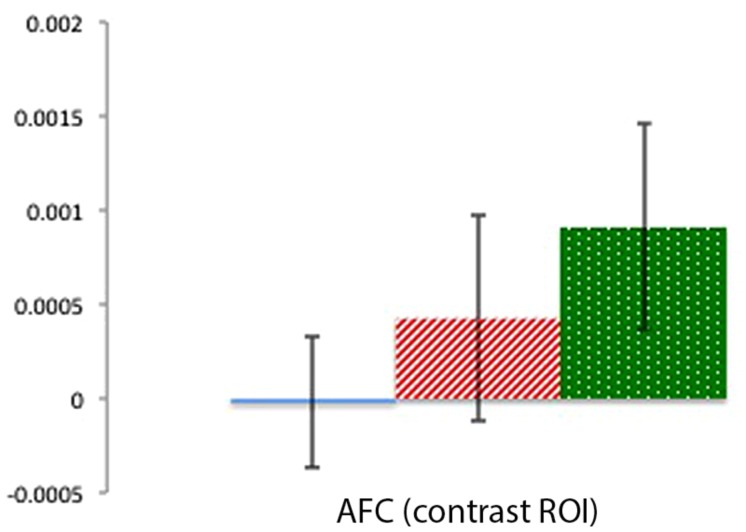
**Percent mean oxy (HbO) signal change for Beliefs conditions (blue solid), Desires conditions (red striped), and Physical conditions (green dotted) in the AFC contrast ROI.** Results indicate a pattern of activation that contrasts with the focal left and right TPJ ROIs.

Mean activation in a center channel over the AFC was examined across the three conditions to provide a contrast for the activation patterns in the focal TPJ ROIs. Contrary to the clear activation differences in TPJ ROIs, there were no significant differences in activation in the AFC region (*t*s < 1.31, *p*s > 0.22). As can be seen in **Figure 6**, this contrast region shows greater signal change for the Physical than for the Mental (beliefs and desires) conditions, which is the reverse of the pattern of activation in the focal TPJ ROIs. To compare brain activity in Beliefs, Desires, and Physical conditions across the three ROIs (LTPJ, RTPJ, and AFC), we conducted a 3 (ROI: LTPJ, RTPJ, AFC) × 3 (Condition: Beliefs, Desires, Physical) repeated-measures ANOVA. A significant main effect of condition confirmed statistical differences across ROIs: *F*(2) = 24.11, *p* < 0.001. Follow up 2 (ROI) × (3 Condition) RM-ANOVAs comparing RTPJ with AFC and LTPJ with AFC also yielded significant main effects of ROI, further confirming that the pattern of activation in the contrast AFC differed from our focal TPJ ROIs [for RTPJ: *F*(1) = 37.901, *p* < 0.001; for LTPJ: *F*(1) = 27.81, *p* < 0.001].

## Discussion

Given that the Beliefs, Desires, and Physical (control) conditions all had the same perceptual and linguistic structure including similar two-part comparisons, differences in neural activation can be attributed to differences in reasoning about the *content* of each condition (i.e., belief-reasoning, desire-reasoning, or reasoning about physical locations) beyond the memory and processing demands common in all conditions. Accordingly, as was clearly demonstrated in our most conservative Group ROI analysis, in both left and especially right TPJ, as predicted, brain activation for belief-reasoning was distinctly greater than brain activation for both physical-reasoning *and*, most focally, desire-reasoning. The beliefs-physical distinction was visible in both left and right TPJ, in line with existing findings that children’s TPJ is recruited for mental-sate reasoning versus reasoning about non-mental information (e.g., Gweon et al., 2012). Critically, participants’ activation for belief-reasoning was significantly greater than *desire*-reasoning for right TPJ, demonstrating a clear, robust belief-specialization on the right. Though visible across analyses for left TPJ, that beliefs-desires distinction reached only marginal significance.

Brain activation over a frontal surface region (AFC) demonstrated a different pattern of activation from the focal TPJ ROIs. Such differential activation patterns in this contrast region indicate that the focal patterns in the right and left TPJ represent brain activity in the temporoparietal cortex, and are not products of systemic noise, or a global activation pattern occurring over the whole head (Boas et al., 2004). Activity in the AFC served only as contrast for our focal TPJ regions, and thus we do not discuss the frontal patterns of activity in depth. However, though the overall reduced signal in the AFC compared to TPJ can be expected given that frontal optodes are generally less sensitive to near-infrared light absorption changes (Cooper et al., 2012), it is possible that such reduced signal also reflects the presence of the default mode network (which exhibits reduced frontal activity during cognitive tasks; Greicius et al., 2002). Future research could explore this possibility.

### Clarifying Neural Correlates of Belief- and Desire-Reasoning

Results of the present study clarify those of the parallel ERP study that used our same neuroimaging task with similar-aged children (Bowman et al., 2012). That ERP study demonstrated that in 7- and 8-years-old, participants’ neural activation for belief-reasoning differentiated from desire-reasoning over right posterior scalp regions. In that study this difference was most prominent when analyses were concentrated on only the trials in which children performed correctly. While, in the present study, the small sample size and block design of the fNIRS study prevented separate analysis of correct trials, our fNIRS data also show differences in brain response for belief- and desire-reasoning, specifically in magnitude of activation. Future fNIRS studies with larger samples that examine only blocks in which children performed correctly may reveal even more robust differences across belief, desire, and non-mental reasoning. It is possible that differences in brain activation are due to differences in computation difficulty across conditions. However, adult ERP research (Liu et al., 2009) using the same task as the present study (and as the parallel child ERP study) suggests otherwise: adults in that parallel ERP study, who critically obtained identical near-perfect accuracy on all three conditions, also showed distinguishable neural responses for belief- versus desire-reasoning at right posterior electrodes. Future, fNIRS studies with older children who are able to achieve similar performance accuracy across conditions, and with modified (e.g., slowed) tasks that foster increased accuracy even at younger ages can shed more light on this question. Larger samples should also be collected to raise power. Nonetheless, as noted, the current results provide findings that are in line with previous ERP (Liu et al., 2009; Bowman et al., 2012) and fMRI research (Saxe et al., 2009; Gweon et al., 2012) on neural correlates of mental-state reasoning.

Beyond replication of ERP results, findings from the present fNIRS study make two additional, critical contributions. First, the increased spatial resolution and unambiguous localization of the fNIRS data demonstrate with high probability that the ‘right posterior’ belief-desire distinction (in ERP data from Bowman et al., 2012) includes regions of specifically the right TPJ. Second, the fNIRS data demonstrate that participants’ activation in right TPJ for belief-reasoning is clearly *greater* than activation for desire-reasoning (compared to ERP results in which differences in waveform amplitude do not necessarily translate to differences in magnitude of neural activity), and further suggest greater activation for belief-reasoning compared to physical-reasoning as well. These additional clarifications help confirm that the pattern of activation in the right TPJ is indeed one of *specialization* for beliefs. Thus, by 6- to 10-years-old, specifically the right TPJ shows a more focused and amplified recruitment of brain substrates for belief-reasoning in particular, beyond non-mental processing, and even beyond processing other mental information such as desires. Perhaps also notable is that plots of the HbO time course for right TPJ reveal, qualitatively, a more pronounced dissociation between Beliefs and Desires conditions toward the end of the block compared to the beginning. Future, research may want to consider this potentially intriguing finding, which could suggest that differences in brain processes for belief- and desire-reasoning emerge only later in the time course.

More speculatively, the present study found initial evidence for possible involvement of the left TPJ in belief-reasoning, though it is important to note that the belief-desires comparison reached only marginal significance there. Several explanations could account for such a finding. It has been argued that neuro-cognitive activations can become more focused and narrowed with development (Casey et al., 2000; but see Poldrack, 2010). And documented changes in networks and connectivity have also been found across development (Fair et al., 2009). One intriguing possibility for the present study is that possibly as children age, belief-specialization becomes more focused to the right TPJ, and left posterior specialization for beliefs diminishes. Indeed, Saxe et al. (2009) measured brain activation (via fMRI) as typically developing 6- through 10-years-old listened to stories describing peoples’ mental states (mental condition), peoples’ interactions and appearances (social condition), and physical objects and scenes (physical condition). Results showed greater activation in the mental condition versus the social condition in *both* the left and right TPJ, but as children aged, specifically the right TPJ was found to increase in selectivity for mental-state processing (in comparison to processing both physical and social stories). A similar pattern of increasing right (but not left) TPJ specialization as children 5–11 years increased in accuracy for mental-state reasoning was found in Gweon et al. (2012). Our fNIRS data and methods—with their increased spatial resolution, clearer measures of individual variation in brain responses, and generally more targeted analytic approach to left TPJ examinations—could have been necessary to reveal the weaker left posterior specialization, whereas the ERP analyses in Bowman et al. (2012) may not have been able to reveal such a left-side effect. Future fNIRS investigations of belief- and desire-reasoning in older children, as well as younger children, and with overall greater sample sizes are important to further explore this intriguing potential developmental effect.

### Broader Implications for ToM Development

Our pattern in which participants’ activations for belief-reasoning clearly exceeded those for desire-reasoning in TPJ has direct implications for understanding the behavioral findings demonstrating that children reach an explicit understanding of desires before they come to an explicit understanding of beliefs (e.g., Wellman and Liu, 2004), pointing to a neural mechanism underlying this progression. That is, our results represent a straightforward developmental possibility that an understanding of beliefs may build on prior desire-understanding, evidenced by additional substrates in right TPJ regions being recruited for specifically belief reasoning—beyond recruitment for desire-reasoning—as belief-understanding becomes more distinct and accurate. Such a developmental scenario builds from behavioral evidence that children progress from an explicit understanding of desires to an explicit understanding of beliefs (e.g., Wellman, 2002; Wellman and Liu, 2004). Indeed, our performance data show that even by middle childhood, children still have greater accuracy on diverse-desires tasks compared to diverse-beliefs tasks (although still younger children, on average, pass diverse-desires and diverse-beliefs tasks, our tasks presented information quickly and thus were more difficult overall)—a pattern consistent with findings demonstrating that belief-understanding can be less accurate/fluent in older children and adults (e.g., Malle, 2004).

## Conclusion

Our findings highlight the utility of fNIRS data for identifying neural specialization in targeted ROI. Beyond providing substantive data of import in its own right, the methods and data we present also provide a platform for future research to address some of the limitations of the present study and to further uncover the neural underpinnings of ToM developments. A critical and obvious limitation is that our sample size is relatively small, thus confirmation of these effects with additional children are needed. Future research may also consider using an HRF modeling based regression approach, as our block-averaging approach may have been more conservative in revealing condition differences given the peak in the HbO curves happened toward the end of the block. Additionally, children of younger ages should be studied. Behaviorally, it is among preschoolers that the biggest differences between desire- versus belief-reasoning are apparent, and it is at this younger age when dramatic differences in explicit belief-reasoning emerge. The child-friendly qualities of fNIRS and the present study design could be usefully employed with these younger children (for whom no current fMRI data are available that examine belief- and desire-reasoning separately, and from whom fMRI data are very difficult to collect).

Even in advance of useful future research, the present study sheds important light on the neural correlates of belief- and desire-reasoning in childhood by pointing to a possible neural mechanism underlying the developmental progression from understanding desires to understanding beliefs evidenced by numerous behavioral studies (e.g., Wellman and Liu, 2004). Specifically, an explicit understanding of beliefs may build off prior understanding of desires, paced by a specialization in the TPJ for reasoning about specifically beliefs (over and above desires). Future research, using fNIRS methods in particular, can now shed further light on how different types of mental states build to form an expert ToM.

## Conflict of Interest Statement

The authors declare that the research was conducted in the absence of any commercial or financial relationships that could be construed as a potential conflict of interest.

## References

[B1] Aslin R. N. (2012). Questioning the questions that have been asked about the infant brain using near-infrared spectroscopy. *Cogn. Neuropsychol.* 29 7–33. 10.1080/02643294.2012.654773 22329690 PMC3461095

[B2] Bartsch K. Wellman H. M. (1995). *Children Talk about the Mind.* New York: Oxford University Press.

[B3] Boas D. A. Dale A. M. Franceschini M. A. (2004). Diffuse optical imaging of brain activation: approaches to optimizing image sensitivity, resolution, and accuracy. *Neuroimage* 23 S275–S288. 10.1016/j.neuroimage.2004.07.011 15501097

[B4] Bowman L. C. Liu D. Meltzoff A. N. Wellman H. M. (2012). Neural correlates of belief- and desire-reasoning in 7- and 8-year-old children: an event-related potential study. *Dev. Sci.* 15 618–632. 10.1111/j.1467-7687.2012.01158.x 22925510 PMC3430978

[B5] Bowman L. C. Wellman H. M. (2014). “Neuroscience contributions to childhood theory-of-mind development,” in *Contemporary Perspectives on Research in Theories of Mind in Early Childhood Education*, ed. Saracho O. N. (Charlotte, NC: Information Age Publishing), 195–224.

[B6] Brink T. T. Urton K. Held D. Kirilina E. Hofmann M. J. Klann-Delius G. (2011). The role of the orbitofrontal cortex in processing empathy stories in 4- to 8-year-old children. *Front. Psychol.* 2:80 10.3389/fpsyg.2011.00080 PMC3110480 21687450

[B7] Carrington S. J. Bailey A. J. (2009). Are there theory of mind regions in the brain? A review of the neuroimaging literature. *Hum. Brain Mapp.* 30 2313–2335. 10.1002/hbm.20671 19034900 PMC6871093

[B8] Casey B. J. Giedd J. N. Thomas K. M. (2000). Structural and functional brain development and its relation to cognitive development. *Biol. Psychol.* 54 241–257. 11035225 10.1016/s0301-0511(00)00058-2

[B9] Castelli F. Happe F. Frith U. Frith C. (2000). Movement and mind: a functional imaging study of perception and interpretation of complex intentional movement patterns. *Neuroimage* 12 314–325. 10.1006/nimg.2000.0612 10944414

[B10] Cooper R. J. Caffini M. Dubb J. Fang Q. Custo A. Tsuzuki D. (2012). Validating atlas-guided DOT: a comparison of diffuse optical tomography informed by atlas and subject-specific anatomies. *Neuroimage* 62 1999–2006. 10.1016/j.neuroimage.2012.05.031 PMC3408558 22634215

[B11] Delpy D. T. Cope M. van der Zee P. Arridge S. Wray S. Wyatt J. (1988). Estimation of optical pathlength through tissue from direct time of flight measurement. *Phys. Med. Biol.* 33 1433–1442. 10.1088/0031-9155/33/12/008 3237772

[B12] Fair D. A. Cohen A. L. Power J. D. Dosenbach N. U. Church J. A. Miezin F. M. (2009). Functional brain networks develop from a “local to distributed” organization. *PLoS Comput. Biol.* 5:e1000381 10.1371/journal.pcbi.1000381 PMC2671306 19412534

[B13] Gagnon L. Yucel M. A. Dehaes M. Cooper R. J. Perdue K. L. Selb J. (2012). Quantification of the cortical contribution to the NIRS signal over the motor cortex using concurrent NIRS-fMRI measurements. *Neuroimage* 15 3933–3940. 10.1016/j.neuroimage.2011.10.054 22036999 PMC3279595

[B14] Gallagher H. L. Happé F. Brunswick N. Fletcher P. C. Frith U. Frith C. D. (2000). Reading the mind in cartoons and stories: an fMRI study of ‘theory of mind’ in verbal and nonverbal tasks. *Neuropsychologia* 38 11–21. 10.1016/S0028-3932(99)00053-6 10617288

[B15] Gopnik A. Slaughter V. (1991). Young children’s understanding of changes in their mental states. *Child Dev.* 62 98–110. 10.2307/1130707

[B16] Gratton G. Brumback C. R. Gordon B. A. Pearson M. A. Low K. A. Fabiani M. (2006). Effects of measurement method, wavelength, and source–detector distance on the fast optical signal. *Neuroimage* 32 1576–1590. 10.1016/j.neuroimage.2006.05.030 16872842

[B17] Greicius M. D. Krasnow B. Reiss A. L. Menon V. (2002). Functional connectivity in the resting brain: a network analysis of the default mode hypothesis. *Proc. Natl. Acad. Sci. U.S.A.* 100 253–258. 10.1073/pnas.0135058100 12506194 PMC140943

[B18] Gweon H. Dodell-Feder D. Bedny M. Saxe R. (2012). Theory of mind performance in children correlates with functional specialization of a brain region for thinking about thoughts. *Child Dev.* 83 1853–1868. 10.1111/j.1467-8624.2012.01829.x 22849953

[B19] Huppert T. J. Diamond S. G. Franceschini M. A. David A. (2009). HomER: a review of time-series analysis methods for near-infrared spectroscopy of the brain. *Appl. Opt.* 48 D280–D298. 10.1364/AO.48.00D280 19340120 PMC2761652

[B20] Kaufman A. S. Kaufman N. L. (1990). *Manual for Kaufman Brief Intelligence Test.* Circle Pines, MN: American Guidance Service.

[B21] Kovelman I. Mascho K. Millott L. Mastic A. Moiseff B. Shalinsky M. (2012). At the rhythm of language: brain bases of language-related frequency perception in children. *Neuroimage* 60 673–682. 10.1016/j.neuroimage.2011.12.066 22230949

[B22] Kovelman I. Shalinsky M. H. Berens M. S. Petitto L. A. (2008). Shining new light on the brain’s “Bilingual Signature.” A functional near infrared spectroscopy investigation of semantic processing. *Neuroimage* 39 1457–1471. 18054251 10.1016/j.neuroimage.2007.10.017 PMC2249758

[B23] Kovelman I. Shalinsky M. H. Berens M. S. Petitto L. A. (2014). Words in the bilingual brain: an fNIRS brain imaging investigation of lexical processing in sign-speech bimodal bilinguals. *Front. Hum. Neurosci.* 8:606 10.3389/fnhum.2014.00606 PMC4139656 25191247

[B24] Kovelman I. Shalinsky M. H. White K. Berens M. S. Schmitt S. Palmer L. (2009). Dual language use in sign-speech bimodal bilinguals using fNIRS Brain-Imaging. *Brain Lang.* 109 112–123. 10.1016/j.bandl.2008.09.008 18976807 PMC2749876

[B25] Kovelman I. Yip J. C. Beck E. L. (2011). Cortical systems that process language, as revealed by non-native speech sound perception. *Neuroreport* 22 947–950. 10.1097/WNR.0b013e32834cdc26 22064664

[B26] Kristen S. Thoermer C. Hofer T. Aschersleben G. Sodian B. (2006). Skalierung von “theory of mind” aufgaben (Scaling of theory of mind tasks). *Z. Entwicklungspsychol. Padagog. Psychol.* 38 186–195. 10.1026/0049-8637.38.4.186

[B27] Liu D. Meltzoff A. N. Wellman H. M. (2009). Neural correlates of belief- and desire-reasoning. *Child Dev.* 80 1163–1171. 10.1111/j.1467-8624.2009.01323.x 19630900 PMC3039678

[B28] Lloyd-Fox S. Richards J. E. Blasi A. Murphy D. G. M. Elwell C. E. Johnson M. H. (2014). Coregistering functional near-infrared spectroscopy with underlying cortical areas in infants. *Neurophotonics* 1 1–16. 10.1117/1.NPh.1.2.025006 PMC4280679 25558463

[B29] Malle B. F. (2004). *How the Mind Explains Behavior: Folk Explanations, Meaning, and Social Interaction.* Cambridge, MA: MIT Press.

[B30] Minagawa-Kawai Y. van der Lely H. Ramus F. Sato Y. Mazuka R. Dupoux E. (2011). Optical brain imaging reveals general auditory and language-specific processing in early infant development. *Cereb. Cortex* 21 254–261. 10.1093/cercor/bhq082 20497946 PMC3020578

[B31] Mosconi M. W. Mack P. B. McCarthy G. Pelphrey K. A. (2005). Taking an “intentional stance” on eye-gaze shifts: a functional neuroimaging study of social perception in children. *Neuroimage* 27 247–252. 10.1016/j.neuroimage.2005.03.027 16023041

[B32] Naito M. Michioka Y. Ozawa K. Ito Y. Kiguchi M. Kanazawa T. (2007). A communication means for totally locked-in als patients based on changes in cerebral blood volume measured with near-infrared light IEICE. *Trans. Inform. Syst.* 90 1028–1037. 10.1093/ietisy/e90-d.7.1028

[B33] Peterson C. C. Wellman H. M. Liu D. (2005). Steps in theory of mind development for children with autism and deafness. *Child Dev.* 76 502–517. 10.1111/j.1467-8624.2005.00859.x 15784096

[B34] Pfeifer J. H. Masten C. L. Borofsky L. A. Dapretto M. Fuligni A. J. Lieberman M. D. (2009). Neural correlates of direct and reflected self-appraisals in adolescents and adults: when social perspective-taking informs self-perception. *Child Dev.* 80 1016–1038. 10.1111/j.1467-8624.2009.01314.x 19630891 PMC3229828

[B35] Poldrack R. A. (2010). Interpreting developmental changes in neuroimaging signals. *Hum. Brain Mapp.* 31 872–878. 10.1002/hbm.21039 20496378 PMC6870770

[B36] Sabbagh M. A. Bowman L. C. Evraire L. E. Ito J. M. B. (2009). Neurodevelopmental correlates of theory of mind in preschool children. *Child Dev.* 80 1147–1162. 10.1111/j.1467-8624.2009.01322.x 19630899

[B37] Saxe R. Kanwisher N. (2003). People thinking about thinking people. The role of the temporo-parietal junction in “theory of mind.” *Neuroimage* 19 1835–1842. 10.1016/S1053-8119(03)00230-1 12948738

[B38] Saxe R. Powell L. J. (2006). It’s the thought that counts: specific brain regions for one component of theory of mind. *Psychol. Sci.* 17 692–699. 10.1111/j.1467-9280.2006.01768.x 16913952

[B39] Saxe R. Wexler A. (2005). Making sense of another mind: the role of the right temporo-parietal junction. *Neuropsychologia* 43 1391–1399. 10.1016/j.neuropsychologia.2005.02.013 15936784

[B40] Saxe R. R. Whitfield-Gabrieli S. Scholz J. Pelphrey K. A. (2009). Brain regions for perceiving and reasoning about other people in school-aged children. *Child Dev.* 80 1197–1209. 10.1111/j.1467-8624.2009.01325.x 19630902

[B41] Shalinsky M. H. Kovelman I. Berens M. S. Petitto L. A. (2009). Exploring cognitive functions in babies, children and adults with near infrared spectroscopy. *J. Vis. Exp.* pii: e1268 10.3791/1268 PMC2780028 19638948

[B42] Sommer M. Meinhardt J. Eichenmüller K. Sodian B. Döhnel K. Hajak G. (2010). Modulation of the cortical false belief network during development. *Brain Res.* 1354 123–131. 10.1016/j.brainres.2010.07.057 20678489

[B43] Strangman G. Culver J. P. Thompson J. H. Boas D. A. (2002). A quantitative comparison of simultaneous BOLD fMRI and NIRS recordings during functional brain activation. *Neuroimage* 17 719–731. 10.1006/nimg.2002.1227 12377147

[B44] Tsuzuki D. Dan I. (2014). Spatial registration for functional near-infrared spectroscopy: from channel position on the scalp to cortical location in individual and group analyses. *Neuroimage* 85 92–103. 10.1016/j.neuroimage.2013.07.025 23891905

[B45] Wellman H. M. (2002). “Understanding the psychological world: developing a theory of mind,” in *Blackwell Handbook of Childhood Cognitive Development*, ed. Goswami U. (Malden, MA: Blackwell Publishing), 167–187.

[B46] Wellman H. M. Fang F. Liu D. Zhu L. Liu G. (2006). Scaling of theory of mind understanding in Chinese children. *Psychol. Sci.* 17 1075–1081. 10.1111/j.1467-9280.2006.01830.x 17201790

[B47] Wellman H. M. Liu D. (2004). Scaling of theory-of-mind tasks. *Child Dev.* 75 523–541. 10.1111/j.1467-8624.2004.00691.x 15056204

[B48] Zhang Q. Brown E. N. Strangman G. E. (2007). Adaptive filtering to reduce global interference in evoked brain activity detection: a human subject case study. *J. Biomed. Opt.* 12:064009 10.1117/1.2804706 18163825

